# Cognitive and Neuropsychiatric Sequelae After SARS-CoV-2 Infection: A Narrative Review and Exploratory Cross-Sectional Study of Neurofilament Light Chain and GFAP

**DOI:** 10.3390/brainsci16030276

**Published:** 2026-02-28

**Authors:** Crystell Guadalupe Guzmán Priego, Jesús Maximiliano Granados Villalpando, Guadalupe del Carmen Baeza Flores, Jorge Luis Ble Castillo, Karla del Socorro Celorio Méndez, Isela Esther Juárez Rojop, Mirian Carolina Martínez López, Sonia Martha López Villarreal, Osvelia Esmeralda Rodríguez Luis, Sergio Quiroz Gómez, Sergio de Jesús Romero Tapia, Jennifer Milagros García Orozco, Wendy Selene López Nácar, Oren Obed Salinas Terrazas, Katia Amairani Jiménez Aragón

**Affiliations:** 1 Cardiometabolism Laboratory, Research Center, Health Sciences Academic Division (DACS), Juarez Autonomous University of Tabasco (UJAT), Villahermosa 86040, Mexico; crystell_guzman@ujat.mx (C.G.G.P.); guadalupe.baeza@ujat.mx (G.d.C.B.F.); karla.celorio@ujat.mx (K.d.S.C.M.); 2 High Specialty Regional Hospital for Mental Health Villahermosa (HRAESMV), Villahermosa 86029, Mexico; 241e13004@alumno.ujat.mx (J.M.G.O.); 241e13003@alumno.ujat.mx (W.S.L.N.); 231e13004@alumno.ujat.mx (O.O.S.T.); 251e13002@alumno.ujat.mx (K.A.J.A.); 3 Metabolic Disease Biochemistry, Research Center, Health Sciences Academic Division (DACS), Juarez Autonomous University of Tabasco (UJAT), Villahermosa 86040, Mexico; jorge.ble@ujat.mx; 4 Lipid Metabolism, Research Center, Health Sciences Academic Division (DACS), Juarez Autonomous University of Tabasco (UJAT), Villahermosa 86040, Mexico; isela.juarez@ujat.mx; 5 Health Sciences Academic Division (DACS), Juarez Autonomous University of Tabasco (UJAT), Villahermosa 86040, Mexico; martinezlopez@hotmail.com (M.C.M.L.); sergio.quiroz@ujat.mx (S.Q.G.); sergio.romero@ujat.mx (S.d.J.R.T.); 6 Microbiology Laboratory, Research Department, Nuevo Leon Autonomous University, Monterrey 64460, Mexico; sonia.lopezvl@uanl.edu.mx (S.M.L.V.); osvelia.rodriguezls@uanl.edu.mx (O.E.R.L.)

**Keywords:** post-COVID-19 condition, long COVID, neuropsychiatry, neurofilament light chain (NfL), glial fibrillary acidic protein (GFAP), cognitive impairment, depression, anxiety, stress, neuroinflammation

## Abstract

**Highlights:**

**What are the main findings?**

- Post-COVID-19 patients exhibit persistent cognitive and neuropsychiatric symptoms independent of clear biomarker correlations.
- Serum neurofilament light chain and GFAP levels were explored without consistent associations with neuropsychiatric symptom severity.

**What are the implications of the main findings?**

- Persistent post-COVID neuropsychiatric symptoms may occur despite the absence of robust neuroglial biomarker alterations.
- Clinical assessment remains essential, while blood-based biomarkers require further validation in post-COVID populations.

**Abstract:**

**Background:** Persistent cognitive and neuropsychiatric symptoms have been increasingly reported as part of the post-COVID-19 condition. Neurofilament light chain (NfL) and glial fibrillary acidic protein (GFAP) are circulating biomarkers of neuronal and astrocytic injury that increase during acute SARS-CoV-2 infection; however, their role in long-term neuropsychiatric sequelae remains unclear. **Objective:** To provide a narrative overview of cognitive and neuropsychiatric sequelae following SARS-CoV-2 infection and to explore the association of plasma NfL and GFAP concentrations with cognitive impairment and neuropsychiatric symptoms in individuals recovered from COVID-19. **Methods:** A narrative review of the literature was conducted, followed by an exploratory cross-sectional study including 41 adults recovered from SARS-CoV-2 infection. Participants were classified according to acute disease severity into two groups. Cognitive function was assessed using MoCA, and neuropsychiatric symptoms were evaluated using DASS-21. Plasma NfL and GFAP concentrations were measured by ELISA. Group comparisons and Spearman correlation analyses were performed. **Results:** A total of 41 individuals were studied; they recovered from moderate or severe COVID-19 and exhibited a higher prevalence of cognitive impairment and neuropsychiatric symptoms compared with those who recovered from mild or asymptomatic infection. Plasma NfL and GFAP concentrations did not differ significantly between severity groups. NfL showed a weak association with the presence of post-COVID-19 condition. **Conclusions:** This study highlights the high burden of persistent cognitive and neuropsychiatric symptoms following moderate and severe SARS-CoV-2 infection. The absence of sustained elevations in circulating NfL and GFAP nearly two years after infection suggests that ongoing symptoms may involve mechanisms beyond overt neuronal or astrocytic injury.

## 1. Introduction

Post-COVID-19 condition, also referred to as long COVID or post-acute sequelae of SARS-CoV-2 infection (PASC), has emerged as a significant public health concern due to the persistence of symptoms many months after initial infection. Among these, cognitive dysfunction (“brain fog”), attention and memory difficulties, and mood disturbances such as depression and anxiety are among the most commonly reported and debilitating manifestations, affecting a substantial proportion of individuals regardless of the severity of their acute illness. Recent large cohort studies have confirmed that memory complaints, concentration problems, and psychiatric symptoms can persist long after recovery from the acute phase of COVID-19 and are often observed independent of pre-existing conditions or hospitalization status [1,2].

Although the mechanisms driving persistent neurological and neuropsychiatric symptoms after SARS-CoV-2 infection remain incompletely understood, accumulating evidence suggests that neuroimmune processes, endothelial dysfunction, and disruption of central nervous system homeostasis may play important roles. A narrative review of long COVID cognitive dysfunction highlights potential contributions from persistent neuroinflammation, blood–brain barrier impairment, and microvascular injury, with associated molecular changes in cytokine signaling and glial function [3]. Similarly, detailed clinical phenotyping has identified immunological disturbances and autonomic dysfunction in individuals with prolonged neurological symptoms, supporting a complex interplay between systemic immune dysregulation and nervous system function [4].

In this context, circulating biomarkers of neuronal injury and astrocytic activation have been investigated as potential indicators of central nervous system involvement in long COVID. Neurofilament light chain (NfL), a marker of axonal integrity, and glial fibrillary acidic protein (GFAP), an intermediate filament protein released with astrocytic stress or reactivity, have been shown to increase during acute SARS-CoV-2 infection, particularly in moderate to severe cases. However, evidence regarding their persistence and relevance to long-term cognitive and neuropsychiatric outcomes is mixed, with some studies reporting normalization of levels months after infection despite ongoing symptoms [5,6].

Several studies have linked elevated levels of these biomarkers to neurological sequelae during acute or early phases of COVID-19, suggesting that neuronal and astrocytic injury may contribute to post-COVID-19 condition in specific contexts [7,8,9,10,11,12,13,14,15,16,17,18,19,20,21,22]. Together, these data support the use of circulating GFAP and NfL as complementary biomarkers of glial and axonal stress in post-COVID-19 neurological disease.

Despite growing evidence of persistent neuropsychiatric and cognitive symptoms following SARS-CoV-2 infection, several gaps remain in the literature. Most studies have focused on the acute or early post-infection phase [15,18,19], whereas data on long-term neuropsychiatric outcomes beyond the first year after infection remain limited [20,22]. In addition, while neurofilament light chain (NfL) and glial fibrillary acidic protein (GFAP) have been extensively studied as markers of acute neuronal and astrocytic injury in COVID-19, their relationship with persistent neuropsychiatric symptoms in the chronic post-COVID-19 phase is not well established. Furthermore, few studies have jointly examined clinical neuropsychiatric outcomes and circulating neuroinjury biomarkers in relatively young populations without major metabolic or psychiatric comorbidities, limiting the understanding of symptom–biomarker dissociation in this context [9,19,20,21].

Therefore, the present exploratory cross-sectional study aimed to characterize long-term neuropsychiatric and cognitive symptoms in individuals with post-COVID-19 condition according to the severity of the acute SARS-CoV-2 infection and to examine their association with plasma NfL and GFAP concentrations nearly two years after infection. We hypothesized that individuals who experienced moderate or severe acute COVID-19 would exhibit a higher burden of persistent neuropsychiatric and cognitive symptoms compared with those with mild or asymptomatic infection, while differences in circulating neuroinjury biomarkers would be less pronounced in the chronic post-infection phase.

Additionally, we hypothesized that NfL and GFAP levels would show limited associations with persistent symptoms, reflecting the contribution of non-structural mechanisms to long-term neuropsychiatric manifestations.

## 2. Materials and Methods

### 2.1. Narrative Review

A narrative review of the literature was conducted to contextualize the exploratory cross-sectional study and to inform its hypotheses. Searches were performed in PubMed and Scopus using terms related to post-COVID-19 condition, neuropsychiatric symptoms, neurofilament light chain, and glial fibrillary acidic protein. Relevant peer-reviewed articles published in English were considered based on their relevance to the study objectives. The review was not intended to be systematic or exhaustive but to provide a conceptual framework supporting the rationale and interpretation of the exploratory analyses.

### 2.2. Study Design and Data Sources

This study employed a cross-sectional, observational, and exploratory analytical design. Data were sourced from primary clinical evaluations and laboratory analyses of participants recruited through a statewide open call. The primary data sources included structured sociodemographic interviews, standardized clinical screening tools for cognitive and neuropsychiatric assessment, and laboratory results from plasma analysis.

### 2.3. Study Setting

The research was conducted at the Research Center of the Academic Division of Health Sciences (DACS) at the Juarez Autonomous University of Tabasco (UJAT) in Villahermosa, Tabasco, Mexico. All clinical procedures, including participant interviews, physical assessments, and biological sample collection, took place within this university-affiliated specialized research facility.

### 2.4. Participants and Procedure

#### 2.4.1. Recruitment and Selection

Participants were recruited via social media dissemination and through institutional announcements and voluntary participation across Villahermosa, Tabasco, Mexico. A non-probabilistic convenience sampling method was utilized due to the lack of comprehensive data on post-COVID patients in Mexico and its states, which made it impossible to define a truly representative sample. Interested individuals attended the research center and were screened based on predefined inclusion and exclusion criteria (see Appendix A) to reduce selection bias as much as possible within the constraints of an exploratory design. It should be noted that no formal sample size calculation was performed, and therefore the sample is not representative of the general population; the findings should be interpreted as preliminary and hypothesis-generating.

#### 2.4.2. Clinical and Laboratory Workflow

1. Screening: Cases meeting inclusion criteria were selected, and relevant clinical variables were recorded.

We conducted the following inclusion (Appendix A.1), exclusion (Appendix A.2) and elimination (Appendix A.3) criteria, grouping them into two different categories:

(a) Severe and Moderate Case Group: Subjects recovered from SARS-CoV-2 infection following hospitalization or the use of immunomodulators.
(b) Mild and asymptomatic Case Group: A group of 10 subjects recovered from asymptomatic or mild SARS-CoV-2 infection. Voluntary donors were selected following an interview to screen for selection criteria.

2. Clinical Assessment: Participants were divided into two groups. A structured questionnaire was administered to collect detailed sociodemographic information (age, sex, education, occupation, marital status), anthropometric data (weight, height, body mass index), and medical history. Family history included neuropsychiatric disorders (e.g., depression, anxiety, post-traumatic stress disorder, multiple sclerosis), chronic non-communicable diseases, and COVID-19–related illness or death among relatives or close contacts. Personal medical history prior to SARS-CoV-2 infection assessed smoking and alcohol use, chronic neurological, psychiatric, and endocrine conditions, as well as regular medication use. COVID-19–related variables included diagnostic testing, number and timing of infections, hospitalization or intensive care admission, treatments received, vaccination status, and the presence of persistent somatic and neuropsychiatric symptoms after recovery. All assessments were conducted during a single study visit by a trained physician using standardized procedures.

All neuropsychiatric and cognitive assessments were administered by trained physicians involved in mental health assessment following standardized procedures. To ensure consistency, all participants were evaluated using the same instruments under identical conditions. All participants were assessed once at a single time point.

3. Sample Collection: Venous blood samples (4 mL) were collected in EDTA-coated (purple top) vacutainer tubes during the same study visit in which clinical and neuropsychiatric assessments were performed. Samples were immediately centrifuged at 3000 rpm for 20 min. The resulting plasma was aliquoted and stored at −80 °C until biochemical analysis.

4. Biochemical Analysis: Plasma samples were processed using a double-antibody sandwich Enzyme-Linked Immunosorbent Assay (ELISA) to determine the concentrations of Neurofilament Light Chain (NfL) (Appendix B.1) and Glial Fibrillary Acidic Protein (GFAP) (Appendix B.2). All assays were performed according to the manufacturer’s instructions.

### 2.5. Measures, Variables, and Data Collection

#### Clinical Instruments

Neuropsychiatric Assessment: Depression Anxiety Stress Scales-21 (DASS-21): This 21-item scale measures depressive symptoms, anxiety symptoms, and stress perception based on the tripartite model of emotion. Cut-off scores are categorized as follows:

- Stress: mild (8–9), moderate (10–12), severe (13–16), extremely severe (≥17).
- Anxiety: mild (4), moderate (5–7), severe (8–9), extremely severe (≥10).
- Depression: mild (5–6), moderate (7–10), severe (11–13), extremely severe (≥14) [23].
- Neuropsychiatric outcomes reflect symptom severity based on standardized self-report instruments and do not represent clinical diagnoses.
- Cognitive Assessment (MoCA): The Montreal Cognitive Assessment (MoCA) is a 30-point screening tool that evaluates eight cognitive domains, including visuospatial/executive function, naming, memory, attention, language, abstraction, delayed recall, and orientation. It has a reported sensitivity of 94% and specificity of 50% for the detection of cognitive impairment. A score of ≤25 was used to indicate cognitive impairment, consistent with previously validated cut-off values for mild cognitive impairment in clinical and research settings. Given its high sensitivity, the MoCA is widely used for screening mild cognitive impairment, particularly in post-infectious and post-acute neurological conditions [24].

The following variables were recorded and operationalized for analysis:

Age, sex, neuropsychiatric personal and family history, post-COVID recovery time, COVID-19 severity (asymptomatic/mild vs. moderate/severe), NfL plasma concentration (ELISA), GFAP plasma concentration (ELISA), DASS-21 scores, severity levels (normal to severe), MoCA score, and post COVID condition as defined by WHO’s Delphi consensus [25].

### 2.6. Statistical Analysis

Data were initially compiled in Microsoft Excel 2021, and all subsequent statistical analyses were performed using IBM SPSS Statistics v26 and GraphPad Prism 9. Distribution normality was evaluated using the Kolmogorov–Smirnov test. Categorical variables were analyzed using chi-square tests, and effect sizes were estimated using odds ratios (OR). Group comparisons for continuous variables were conducted using Student’s *t*-test for normally distributed variables or the Mann–Whitney U test for non-normally distributed variables, as appropriate. Correlation analyses were performed using Spearman’s rank correlation coefficient. Although age showed a normal distribution, it was treated as a descriptive variable and was not included in correlation analyses; therefore, Pearson’s correlation coefficient was not applied.

## 3. Results

### 3.1. Description of the Study Population

The study group consisted of 41 subjects recovered from SARS-CoV-2 infection who met the inclusion criteria. The study population comprised 24 (58.53%) women with a mean age of 24.38 ± 8.75 years and 17 (41.46%) men with a mean age of 28.29 ± 11.1 years.

The first group, consisting of patients recovered from moderate or severe SARS-CoV-2 infection, accounted for 75.61% (*n* = 31) of the sample, of which 32.25% (*n* = 10) were men and 67.74% (*n* = 21) were women. The second group, comprising patients recovered from mild or asymptomatic SARS-CoV-2 infection, represented 24.39% (*n* = 10) of the subjects, with 70% (*n* = 7) being men and 30% (*n* = 3) women. A statistically significant difference in sex distribution was observed between the groups (*p* = 0.035; Table 1).

### 3.2. General Sociodemographic Characteristics of the Sample

Table 1 presents the sociodemographic characteristics of the study population. The mean age was 26 ± 9.85 years. The majority of participants were students (27, 65.9%), held or were pursuing an undergraduate degree (37, 90.2%), and were single (35, 85.4%). No statistically significant differences were found in the distribution of age, occupation, education level, or marital status between the groups.

### 3.3. Characteristics of SARS-CoV-2 Infection Recovery

The mean time elapsed since recovery from SARS-CoV-2 infection was 22.73 ± 11.95 months, ranging from 4 to 41 months. The moderate/severe recovery group (Group 1) had a mean of 22.64 ± 12.17 months, while the mild/asymptomatic group (Group 2) had a mean of 23 ± 11.86 months. Student’s *t*-test showed no statistically significant difference between the groups (t = −0.081, *p* = 0.93).

Regarding post-COVID conditions, 11 subjects reported persistent symptoms. Of these, 10 (90.91%) belonged to the moderate/severe recovery group, while only 1 (9.09%) belonged to the mild/asymptomatic group.

### 3.4. Cognitive Impairment, Depressive Symptoms, Anxiety Symptoms, and Stress Perception

#### 3.4.1. Cognitive Impairment

The mean MoCA score was 24.46 ± 3.12 out of 30 possible points. Using a cutoff of 26, the sample showed average cognitive impairment, with 51.21% (*n* = 21) scoring below the threshold.

Cognitive impairment was present in 64.51% (*n* = 20) of patients in the moderate/severe group, compared to only 10% (*n* = 1) in the mild/asymptomatic group. Chi-square analysis yielded a significant result (χ^2^ = 9.994, *p* = 0.003), with an Odds Ratio (OR) of 16.36 (95% CI: 1.82–146.66). Mean MoCA scores were significantly lower in Group 1 (23.71 ± 3.04) compared to Group 2 (26.8 ± 2.09) (t = −2.976, *p* = 0.005).

#### 3.4.2. Depressive Symptoms

The mean depressive symptoms score was 7.07 ± 5.87 out of 21 points, indicating the presence of depressive symptoms in the sample (56.09%, *n* = 23). Prevalence was 74.19% (*n* = 23) in the moderate/severe group, while no cases were reported in the mild/asymptomatic group (χ^2^ = 16.9, *p* < 0.001, OR = 3.87, 95% CI: 2.13–7.03).

Mean scores were significantly higher in moderate/severe cases (9 ± 5.45) than in mild/asymptomatic cases (1.1 ± 1.37) (*p* < 0.001). Severe or extremely severe depressive symptoms were found in 12 cases (38.70%), all within the moderate/severe group (χ^2^ = 5.473, *p* = 0.019, OR = 1.632, 95% CI: 1.233–2.158; Table 2).

#### 3.4.3. Anxiety Symptoms

The mean anxiety symptoms score was 8.2 ± 5.27 points, with 70.73% (*n* = 29) of the sample presenting anxiety symptoms. Prevalence was 87.09% (*n* = 27) in the moderate/severe group versus 20% (*n* = 2) in the mild/asymptomatic group (χ^2^ = 16.44, *p* < 0.001, OR = 27, 95% CI: 4.15–175.49).

Mean scores were significantly higher in moderate/severe cases (10.06 ± 4.52) than in mild/asymptomatic cases (2.4 ± 2.4) (*p* < 0.001). Severe or extremely severe anxiety symptoms were present in 22 subjects (70.96%), all from the moderate/severe group (χ^2^ = 15.314, *p* < 0.001, OR = 3.444, 95% CI: 1.987–5.972; Table 3).

#### 3.4.4. Stress Perception

The mean stress perception score was 10.05 ± 5.42 points, with 70.73% (*n* = 29) of the sample presenting stress perception. Prevalence was 87.09% (*n* = 27) in the moderate/severe group versus 20% (*n* = 2) in the mild/asymptomatic group (χ^2^ = 16.44, *p* < 0.001, OR = 27, 95% CI: 4.15–175.49).

Mean scores were significantly higher in moderate/severe cases (11.87 ± 4.71) than in mild/asymptomatic cases (4.4 ± 3.13) (*p* < 0.001). Severe or extremely severe stress perception was present in 16 subjects (51.61%), all from the moderate/severe group (χ^2^ = 8.465, *p* = 0.004, OR = 2.067, 95% CI: 1.437–2.973; Table 4).

A significantly higher prevalence of cognitive impairment, depressive symptoms, anxiety symptoms, and stress perception was observed in the moderate/severe recovery group compared to the mild/asymptomatic group (*p* < 0.005 in all cases; Table 5).

### 3.5. Concentrations of Neurofilament Light Chain and Glial Fibrillary Acidic Protein

**Figure 1:**
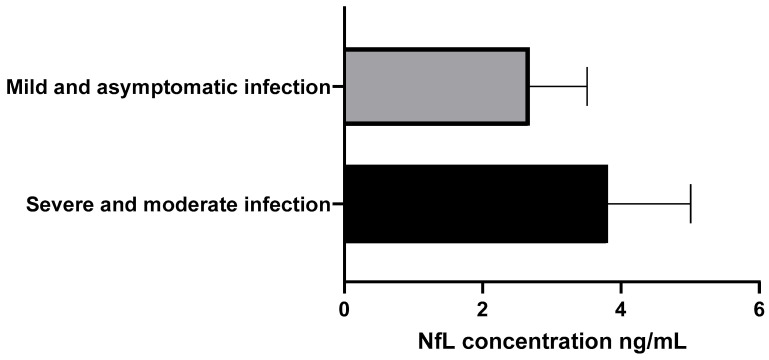
NfL Concentration in ng/mL. Group 1: Moderate and severe infection. Group 2: Mild/asymptomatic infection.

NfL concentrations were evaluated by severity: the moderate/severe group had a mean of 3.81 ± 1.2 ng/mL, while the mild/asymptomatic group had a mean of 2.68 ± 0.83 ng/mL. Median NfL concentration was 1.75 ng/mL, IQR 3.3 ng/mL (Figure 1). The Mann–Whitney U test showed no significant difference (Z = 0.515, *p* = 0.61, 95% CI: −3.29 to 5.55).

**Figure 2:**
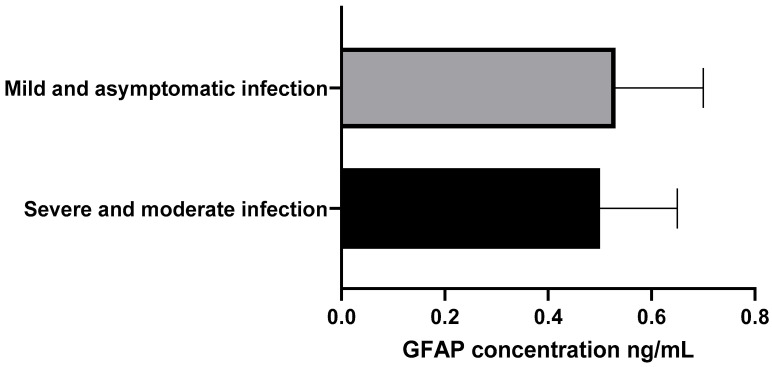
GFAP Concentration in ng/mL. Group 1: Moderate and severe infection. Group 2: Mild/asymptomatic infection.

GFAP concentrations were also evaluated: the moderate/severe group had a mean of 0.5 ± 0.15 ng/mL, and the mild/asymptomatic group had a mean of 0.53 ± 0.17 ng/mL. Median GFAP concentration was 0.51 ng/mL, IQR 0.2 ng/mL. The Mann–Whitney U test showed no significant difference (Z = 0.456, *p* = 0.65, 95% CI: −0.142 to 0.089) (Figure 2).

### 3.6. Protein Concentrations and Clinical Characteristics: Correlation Analysis

A Kolmogorov–Smirnov test indicated a non-normal distribution; therefore, non-parametric tests were used. A moderate correlation was found between NfL and GFAP concentrations (rho = 0.421, *p* = 0.006) and between post-COVID conditions and female sex (rho = 0.51, *p* = 0.001).

Low correlations were observed between COVID-19 severity and female sex (rho = 0.329, *p* = 0.036) and between plasma NfL concentrations and the development of post-COVID conditions (rho = 0.307, *p* = 0.05; Table 6).

Spearman’s Rho also revealed moderate correlations between severe stress perception and severe anxiety symptoms (rho = 0.543, *p* < 0.001), severe anxiety symptoms and severe depressive symptoms (rho = 0.49, *p* = 0.001), and severe depressive symptoms and severe stress perception (rho = 0.474, *p* = 0.002; Table 7).

## 4. Discussion

Neuroimmune dysregulation has been proposed as a central mechanism underlying the neurological and psychiatric manifestations of post-COVID-19 condition [7,26,27]. Acute SARS-CoV-2 infection induces a marked systemic inflammatory response characterized by elevated cytokines, chemokines, and immune cell activation, which has been proposed to disrupt blood–brain barrier integrity and promote glial activation, potentially altering neuronal homeostasis [28,29,30]. These processes may lead to long-lasting changes in neural network function even after systemic inflammation has resolved and in the absence of ongoing structural neuronal injury.

Several proteins released into cerebrospinal fluid and blood during neuronal or astrocytic stress have been widely used as indirect indicators of central nervous system involvement in COVID-19 and other neuroinflammatory or neurodegenerative conditions [31,32]. These include neurofilament light, medium, and heavy chains, which reflect axonal integrity; glial fibrillary acidic protein (GFAP), a marker of astrocytic reactivity and injury; as well as tau and phosphorylated tau, S100B, TNF-α, and vimentin, which together capture different aspects of neuronal damage, glial activation, and blood–brain barrier disruption [26,27,31,32,33,34,35,36,37,38,39].

The present study focused on the plasma proteins neurofilament light chain (NfL) and glial fibrillary acidic protein (GFAP), two well-established biomarkers of axonal and astrocytic integrity, respectively. NfL is released into biofluids in response to axonal stress or injury, whereas GFAP reflects astrocytic reactivity and damage. Both proteins increase during acute neurological injury and have been reported to rise during moderate and severe SARS-CoV-2 infection, reflecting transient central nervous system involvement [7,8,26,27,34,35,36,37,38].

Accordingly, this study aimed to explore whether long-term plasma concentrations of NfL and GFAP are associated with the severity of acute SARS-CoV-2 infection and with persistent cognitive impairment, depressive symptoms, anxiety symptoms, and stress perception in individuals recovered from COVID-19.

The present study identified a weak but statistically significant association between plasma NfL concentrations and the presence of post-COVID-19 condition, as well as a moderate correlation between NfL and GFAP levels. These findings are biologically plausible given that both proteins reflect complementary aspects of neuronal and astrocytic stress during acute SARS-CoV-2 infection and tend to rise in parallel during periods of central nervous system involvement.

The absence of sustained group-level elevation of either biomarker nearly two years after infection is consistent with previous reports showing that NfL and GFAP typically normalize within months after the acute phase, even in patients who go on to develop persistent neurological or psychiatric symptoms. Together, these results suggest that while transient axonal and astrocytic injury may occur during acute COVID-19, long-term post-COVID-19 symptomatology is not driven by ongoing large-scale structural neuronal damage detectable by these circulating biomarkers [38,39,40,41].

In this study, cognitive impairment was highly prevalent among participants who had experienced moderate or severe COVID-19. Acute disease severity showed an association with long-term cognitive impairment, although the wide confidence interval of the OR reflects the small size and imbalance of the comparison groups. These findings are consistent with multiple previous studies reporting an association between COVID-19 severity, neurological involvement, and subsequent cognitive dysfunction [39,40,41,42,43,44,45,46,47,48].

Similarly, symptoms of depression, anxiety, and stress perception were markedly more frequent among individuals recovered from moderate or severe SARS-CoV-2 infection. Moderate or severe COVID-19 was associated with significantly increased odds of depressive symptoms, anxiety symptoms, and stress perception, suggesting a greater long-term neuropsychiatric burden in this population. MoCA scores significantly differed between groups, with lower scores in the moderate/severe group indicating greater cognitive impairment. Similarly, DASS-21 scores were significantly higher in these subjects, reflecting a greater burden of depressive symptoms, anxiety symptoms, and stress perception.

No significant association was observed between plasma NfL or GFAP concentrations and the severity of acute SARS-CoV-2 infection, in contrast with other studies [28,29,30,49]. While this finding may partly reflect methodological considerations, it is also biologically plausible given the long interval between infection and evaluation in our cohort (mean 22.7 months) [40,47]. Both NfL and GFAP are known to rise during acute and subacute neuronal and astrocytic injury and typically normalize within months after the inflammatory phase of COVID-19 has resolved [19].

An important implication of these findings is the dissociation between persistent neuropsychiatric symptoms and circulating markers of structural neuronal and astrocytic injury. Despite the high prevalence of cognitive impairment, depressive symptoms, anxiety symptoms, and stress perception observed in individuals who experienced moderate or severe COVID-19, plasma NfL and GFAP concentrations were largely within ranges expected for healthy adults nearly two years after infection. Their normalization in this cohort therefore suggests the absence of ongoing large-scale axonal or astrocytic structural damage nearly two years after infection.

In this sense, while acute disease severity showed a strong association with long-term cognitive and neuropsychiatric outcomes in this study, it does not necessarily imply persistent neurodegeneration [28,48].

Several additional associations were identified in exploratory analyses. Female sex was significantly associated with the presence of post-COVID-19 condition, greater acute disease severity, and higher plasma concentrations of NfL and GFAP. These findings are consistent with a growing body of literature reporting a higher prevalence of post-COVID-19 condition in women and suggesting sex-specific vulnerability to immune-mediated and neurobiological sequelae following SARS-CoV-2 infection [28,29,50,51,52,53,54,55].

Increasing evidence indicates that post-COVID-19 symptoms may arise from lasting alterations in immune–brain signaling, glial priming, synaptic function, or network-level dysregulation that are not captured by circulating markers of structural injury such as NfL and GFAP [28,30,49].

This pattern suggests that long-term post-COVID-19 neurological and psychiatric sequelae are not driven by ongoing large-fiber axonal degeneration or astrocytic destruction, but rather by more subtle and potentially reversible alterations in neuroimmune signaling, glial function, synaptic organization, and large-scale brain network dynamics. Such a dissociation between symptoms and classical neuroinjury biomarkers has been reported in other post-infectious and neuroimmune conditions and may represent a defining biological feature of post-COVID-19 condition [5,28,30,49].

Moreover, only a subset of participants fulfilled criteria for post-COVID-19 condition at the time of assessment. The weak association observed between NfL and post-COVID-19 status may reflect subtle or intermittent axonal stress in a subgroup of patients, but it does not account for the overall burden of cognitive and emotional symptoms observed in those with prior moderate or severe infection.

As expected, strong correlations were observed among depressive symptoms, anxiety, and stress perception severity, reflecting the close clinical and neurobiological coupling of affective and stress-related symptoms in post-COVID-19 condition.

Age was also correlated with NfL and GFAP concentrations, consistent with prior studies showing that these biomarkers increase with aging [7,8,9,10,17]. Although no significant age differences were observed between the severity groups in this cohort, age-related biomarker variability may still contribute to interindividual differences and should be accounted for in future, larger studies. Also, the relatively narrow age range of the study population and the absence of pre-COVID-19 baseline measurements limit the ability to perform age-adjusted analyses or to exclude subtle chronic neuroaxonal or astrocytic alterations.

An additional interpretation of these findings is that the severity of the acute SARS-CoV-2 infection may act as a biological “imprinting” event on the central nervous system, setting long-term trajectories of neuroimmune and network dysfunction that persist after the resolution of overt tissue injury. Severe systemic inflammation, hypoxia, endothelial dysfunction, and cytokine surges during the acute phase may induce durable changes in microglial state, astrocytic signaling, synaptic pruning, and blood–brain barrier regulation, which in turn affect cognition, mood, and stress regulation. Such mechanisms may help account for the observed association between acute disease severity and long-term neuropsychiatric outcomes in this cohort, even though circulating markers of axonal and astrocytic structural damage have returned to baseline.

These findings also have important clinical implications. The dissociation between persistent neuropsychiatric symptoms and circulating markers of neuronal and astrocytic injury suggests that routine neurodegeneration biomarkers may have limited utility for identifying or monitoring patients with post-COVID-19 neurological and psychiatric sequelae. Instead, clinical assessment, neuropsychological testing, and potentially functional or immune-based biomarkers may be more informative for guiding patient care. This underscores the need to conceptualize post-COVID-19 condition as a disorder of brain function and immune–brain interaction rather than as a progressive neurodegenerative process, with direct implications for the development of targeted therapeutic and rehabilitative strategies.

Furthermore, while depressive symptoms, anxiety symptoms, and stress perception symptoms were highly prevalent in this cohort, these symptoms should not be interpreted as purely psychological or secondary to emotional distress alone. The strong association between acute COVID-19 severity and long-term cognitive and affective outcomes, together with the consistency of these findings across independent studies, supports a biological contribution to post-COVID-19 neuropsychiatric sequelae. Immune-mediated alterations of neural circuits involved in cognition, mood regulation, and stress processing provide a plausible mechanistic substrate linking systemic viral illness to persistent psychiatric and cognitive symptoms, distinguishing post-COVID-19 condition from primary psychiatric disorders.

In this sense, although neuroimmune dysregulation has been implicated in post-COVID-19 condition, the present study focused on established markers of neuronal and astrocytic injury; inflammatory biomarkers were beyond the scope of this exploratory design.

It is also important to recognize that NfL and GFAP capture only specific aspects of central nervous system pathology, namely large-fiber axonal injury and astrocytic structural damage. They do not reflect synaptic dysfunction, microglial activation, neurotransmitter imbalance, or alterations in functional connectivity, all of which are increasingly implicated in post-COVID-19 neurological and psychiatric symptoms. The absence of sustained elevations in these biomarkers should therefore not be interpreted as evidence of an absence of central nervous system involvement, but rather as an indication that the dominant pathological processes in post-COVID-19 condition may lie beyond the scope of currently available circulating structural injury markers.

This study has several limitations that should be considered when interpreting the findings. The sample size was modest, reflecting the logistical complexity and cost of comprehensive neuropsychological and biomarker assessments, as well as the stringent exclusion criteria applied to minimize confounding by metabolic and psychiatric comorbidities. While this approach strengthened internal validity, it limited statistical power and generalizability to the broader post-COVID-19 population, in which such comorbidities are common, particularly for biomarker analyses.

Additionally, the cross-sectional design and modest sample size limited the ability to account for multiple lifestyle and psychosocial factors that may influence neuropsychiatric outcomes over time, including sleep quality, academic or occupational stress, interpersonal relationships, and emotional state. These unmeasured variables may have contributed to interindividual variability and should be addressed in future longitudinal studies. Finally, the absence of pre-COVID-19 baseline assessments of neuropsychiatric symptoms and cognitive performance precludes causal inferences regarding symptom onset and changes over time.

The sex distribution differed between severity groups, with a higher proportion of women in the moderate/severe group. Given that female sex was also associated with post-COVID-19 condition and with higher NfL and GFAP concentrations, residual confounding by sex cannot be excluded and may have influenced both symptom prevalence and biomarker variability. Future studies with sex-balanced cohorts and multivariable adjustment will be required to clarify these relationships.

In addition, participants were evaluated a mean of nearly two years after their acute SARS-CoV-2 infection. This long interval likely contributed to the normalization of circulating NfL and GFAP concentrations and precludes conclusions about biomarker dynamics during the acute and subacute phases of the disease.

The cross-sectional design further limits causal inference, as no baseline or acute-phase biomarker measurements were available for within-subject comparison. Longitudinal studies beginning during acute infection and extending into long-term follow-up will be required to clarify the temporal relationship between neuroaxonal and astrocytic injury and the development of persistent cognitive and neuropsychiatric symptoms. Furthermore, not having direct support from the hospitals involved in the treatment of patients, there was no direct access to their records; therefore, the information collected is dependent on the interviewee.

Furthermore, several methodological limitations related to biomarker quantification should be acknowledged. First, plasma NfL and GFAP concentrations were measured using conventional sandwich ELISA, which has lower analytical sensitivity compared with ultrasensitive platforms such as single-molecule array (SIMOA), particularly for detecting subtle or chronic neuroaxonal injury in young populations. Second, biomarker levels were assessed at a single time point nearly two years after infection, which may have limited the ability to capture transient or earlier elevations. Finally, the absence of pre-COVID-19 baseline measurements limits the ability to draw definitive conclusions regarding individual-level changes over time.

Future studies should employ larger, prospectively followed cohorts to increase statistical power and enable stratification by acute disease severity, sex, and post-COVID-19 clinical phenotype. Longitudinal designs with biomarker and neuropsychological assessments during acute infection and at multiple post-recovery time points will be essential to characterize the temporal dynamics of neuronal and glial injury and their relationship to the development and persistence of cognitive and neuropsychiatric symptoms.

The incorporation of viral variant typing and detailed clinical phenotyping may further clarify heterogeneity in post-COVID-19 trajectories, as different variants and host responses may differentially influence neurological outcomes. Expanding biomarker panels to include additional indicators of neuronal injury, astrocytic activation, immune signaling, and blood–brain barrier integrity, such as tau, phosphorylated tau, S100B, and inflammatory cytokines, would provide a more comprehensive picture of central nervous system involvement.

Collaboration with hospitals, outpatient clinics, and diagnostic laboratories will be critical to facilitate recruitment of well-characterized cohorts and to support the integration of biomarker, clinical, and neuropsychological data at scale.

Taken together, these exploratory findings are consistent with the conceptualization of post-COVID-19 condition as a disorder of brain network regulation rather than one of ongoing structural neurodegeneration. Cognitive, emotional, and stress-related symptoms are mediated by distributed cortico–limbic and brainstem networks that are highly sensitive to immune signaling, glial modulation, and autonomic input. Disruption of these networks during acute SARS-CoV-2 infection may lead to persistent dysregulation of attention, mood, and stress responses without requiring ongoing neuronal loss. This framework is consistent with the normal or near-normal levels of NfL and GFAP observed in this cohort and aligns post-COVID-19 condition with other post-infectious and neuroimmune syndromes characterized by prominent symptoms in the absence of overt tissue destruction.

## 5. Conclusions

Individuals who experienced moderate or severe SARS-CoV-2 infection showed a higher prevalence of persistent cognitive impairment and affective symptoms compared with those with mild or asymptomatic disease. These findings suggest that greater acute disease severity may be associated with an increased risk of long-term neuropsychiatric sequelae; however, given the cross-sectional design, limited sample size, and absence of pre-pandemic baseline data, causality cannot be inferred. Nearly two years after infection, plasma NfL and GFAP concentrations did not differ between severity groups, indicating no detectable ongoing axonal or astrocytic injury at the group level. The weak association between NfL and post-COVID-19 condition should be interpreted cautiously and may reflect subtle neuroaxonal stress in a subset of individuals. Future longitudinal studies with larger samples and pre-infection measures are warranted to clarify these associations.

## Figures and Tables

**Table 1:** Sociodemographic characteristics: age, occupation, education, and marital status.

Characteristics	Total (*n* = 41)	Moderate/Severe (*n* = 31)	Mild/Asymptomatic (*n* = 10)	Statistics
Sex				
- Men	17 (41.46%)	10 (32.25%)	7 (70%)	χ^2^ = 4.437, *p* = 0.035 *
- Women	24 (58.53%)	21 (67.74%)	3 (30%)	
**Age**	26 ± 9.856	26.58 ± 10.44	24.2 ± 7.983	t = 0.659, *p* = 0.513
**Occupation**				χ^2^ = 1.23, *p* = 0.53
- Student	27 (65.85%)	21 (67.74%)	6 (60%)	
- Salaried	12 (29.26%)	8 (25.80%)	4 (40%)	
- Self-employed	2 (4.87%)	2 (6.45%)	0	
**Education**				χ^2^ = 1.43, *p* = 0.23
- Undergraduate	37 (90.24%)	27 (87.09%)	10 (100%)	
- Postgraduate	4 (9.75%)	4 (12.90%)	0	
**Marital Status**				χ^2^ = 2.26, *p* = 0.519
- Single	35 (85.36%)	25 (80.54%)	10 (100%)	
- Married	3 (7.31%)	3 (9.67%)	0	
- Domestic Partnership	2 (4.87%)	2 (6.45%)	0	
- Divorced	1 (2.44%)	1 (3.22%)	0	

* *p* < 0.05.

**Table 2:** Distribution of depressive symptoms, severe depressive symptoms, and extremely severe depressive symptoms.

Presence of	Total (*n* = 41)	Moderate/Severe (*n* = 31)	Mild/Asymptomatic (*n* = 10)	Chi-Square	OR (95% CI)
Depressive symptoms	23 (56.09%)	23 (74.19%)	0 (0%)	χ^2^ = 16.9, *p* < 0.001 **	3.87 (2.13–7.03)
Severe/extremely severe depressive symptoms	12 (29.26%)	12 (38.70%)	0 (0%)	χ^2^ = 5.473, *p* = 0.019 *	1.632 (1.233–2.158)

* *p* < 0.05, ** p < 0.005.

**Table 3:** Distribution of anxiety symptoms, severe anxiety symptoms, and extremely severe anxiety symptoms.

Presence of	Total (*n* = 41)	Moderate/Severe (*n* = 31)	Mild/Asymptomatic (*n* = 10)	Chi-Square	OR (95% CI)
Anxiety symptoms	29 (70.73%)	27 (87.09%)	2 (20%)	χ2 = 16.44, *p* < 0.001 **	27 (4.15–175.49)
Severe/extremely severe anxiety symptoms	22 (53.65%)	22 (70.96%)	0 (0%)	χ2 = 15.314, *p* < 0.001 **	3.444 (1.987–5.972)

** *p* < 0.005.

**Table 4:** Distribution of stress perception, severe stress perception, and extremely severe stress perception.

Presence of	Total (*n* = 41)	Moderate/Severe (*n* = 31)	Mild/Asymptomatic (*n* = 10)	Chi-Square	OR (95% CI)
Stress perception	29 (70.73%)	27 (87.09%)	2 (20%)	χ2 = 16.44, *p* < 0.001 **	27 (4.15–175.49)
Severe/extremely severe stress perception	16 (39.02%)	16 (51.61%)	0 (0%)	χ2 = 8.465, *p* = 0.004 **	2.067 (1.437–2.973)

** *p* < 0.005.

**Table 5:** Summary distribution of cognitive impairment, depressive symptoms, anxiety symptoms, and stress perception.

Presence of	Total (*n* = 41)	Moderate/Severe (*n* = 31)	Mild/Asymptomatic (*n* = 10)	Chi-Square	OR (95% CI)
Cognitive Impairment	21 (51.21%)	20 (64.51%)	1 (10%)	χ2 = 9.99, *p* = 0.003 **	16.36 (1.82–146.66)
Depressive symptoms	23 (56.09%)	23 (74.19%)	0 (0%)	χ2 = 16.9, *p* < 0.001 **	3.87 (2.13–7.03)
Anxiety symptoms	29 (70.73%)	27 (87.09%)	2 (20%)	χ^2^ = 16.44, *p* < 0.001 **	27 (4.15–175.49)
Stress perception	29 (70.73%)	27 (87.09%)	2 (20%)	χ^2^ = 16.44, *p* < 0.001 **	27 (4.15–175.49)

** *p* < 0.005.

**Table 6:** Correlation between protein concentrations, post-COVID conditions, severity, and sex.

Correlation	NfL	GFAP	Post-COVID Conditions	Severity	Female Sex
NfL	-	0.421 (0.006) *	0.307 (0.05) *	−0.041 (0.8)	−0.061 (0.706)
GFAP	0.421 (0.006) *	-	0.007 (0.965)	−0.094 (0.56)	−0.006 (0.969)
Post-COVID	0.307 (0.05) *	0.007 (0.965)	-	0.216 (0.176)	0.510 (0.001) **
Severity	−0.041 (0.8)	−0.094 (0.56)	0.216 (0.176)	-	0.329 (0.036) *
Female Sex	−0.061 (0.706)	−0.006 (0.969)	0.510 (0.001) **	0.329 (0.036) *	-

* *p* < 0.05, ** *p* < 0.005 (Spearman’s Rho).

**Table 7:** Correlation between protein concentrations, severe depressive symptoms, severe anxiety symptoms, severe stress perception, and cognitive impairment.

Correlation	NfL	GFAP	Severe Depressive Symptoms	Severe Anxiety Symptoms	Severe Stress Perception
NfL	-	0.421 (0.006) *	0.077 (0.632)	0.079 (0.625)	0.079 (0.625)
GFAP	0.421 (0.006) *	-	−0.082 (0.612)	−0.014 (0.928)	0.027 (0.865)
Severe Dep.	0.077 (0.632)	−0.082 (0.612)	-	0.49 (0.001) **	0.474 (0.002) **
Severe Anx.	0.079 (0.625)	−0.014 (0.928)	0.49 (0.001) **	-	0.543 (0.000) **
Severe Stress	0.079 (0.625)	0.027 (0.865)	0.474 (0.002) **	0.543 (0.000) **	-
Cog. Impair.	0.024 (0.881)	−0.119 (0.46)	−0.123 (0.44)	0.267 (0.091)	−0.02 (0.9)

* *p* < 0.05, ** *p* < 0.005.

## Data Availability

The data that support the findings of this study are available from the corresponding author upon reasonable request due to privacy and ethical restrictions. The datasets contain sensitive clinical and neuropsychiatric information, and public sharing could compromise participant confidentiality. Access to the data may be granted following appropriate ethical review and in accordance with institutional and national regulations.

## Abbreviations

The following abbreviations are used in this manuscript:

COVID	Coronavirus disease
GFAP	Glial Fibrillary Acidic Protein
NfL	Neurofilament light chain
SARS	Severe Acute Respiratory Syndrome

## Appendix A

### Appendix A.1. Inclusion Criteria

- Age between 18 and 60 years.
- Completion of all sections of the research instrument.
- Signed informed consent.

(A) Severe and Moderate Case Group:
  - Diagnosis of SARS-CoV-2 infection confirmed by rtPCR.
  - Clinical data indicating severe or moderate SARS-CoV-2 infection (hospitalization, admission to a COVID-19 care center, use of immunomodulators such as corticosteroids, calcineurin antagonists, or monoclonal antibodies, dyspnea at rest or upon minimal exertion, intubation, assisted mechanical ventilation, or oxygen saturation below 92%).

(B) Mild/Asymptomatic Group: Asymptomatic subjects or those recovered from mild or asymptomatic SARS-CoV-2 infection:
  - Diagnosis of COVID-19 confirmed by rtPCR.
  - Clinical data pertinent to mild or asymptomatic COVID-19 (optimal oxygen saturation, absence of severe symptoms and/or signs).

### Appendix A.2. Exclusion Criteria

- Presence of systemic diseases associated with increased GFAP and/or NfL levels: Obesity (BMI > 30), systemic arterial hypertension, Type 1 and Type 2 diabetes mellitus, fibromyalgia, Crohn’s disease, ulcerative colitis, autoimmune vasculitis, and systemic lupus erythematosus.
- Presence of neurodegenerative diseases: Multiple sclerosis, Amyotrophic Lateral Sclerosis (ALS), Alzheimer’s disease, and Parkinson’s disease.
- Psychiatric illness prior to SARS-CoV-2 infection.
- Chronic alcoholism, smoking, or other substance use disorders.

### Appendix A.3. Elimination Criteria

- Hemolysis of blood sample.
- Coagulation of the blood sample.
- Lipemic samples.

## Appendix B

### Appendix B.1. NfL Quantification Protocol

The Human Neurofilament-Light Chain (NfL) ELISA Kit (MyBioSource, San Diego, CA, USA) was stored at 2–8 °C. Performance characteristics were as follows: detection range 0.1–35 ng/mL, sensitivity 0.054 ng/mL (approximately 54 pg/mL), intra-assay coefficient of variation (CV) < 8%, and inter-assay CV < 10%.

The assay was performed according to the manufacturer’s instructions. Plates, reagents, and samples were brought to room temperature prior to analysis. Blank, standard (S1–S6), and sample wells were established. Subsequently, 50 μL of standards or samples were added to the respective wells, followed by the addition of 100 μL of horseradish peroxidase (HRP) conjugate to all wells except blanks. Plates were incubated at 37 °C for 60 min and then washed eight times with 175 μL of wash buffer. Next, 50 μL of Chromogen A and 50 μL of Chromogen B were added to each well and incubated at 37 °C for 15 min. The reaction was stopped by adding 50 μL of stop solution, and optical density was measured at 450 nm using a spectrophotometer within 15 min. All samples and standards were analyzed in duplicate.

### Appendix B.2. GFAP Quantification Protocol

The Human Glial Fibrillary Acidic Protein (GFAP) ELISA Kit (MyBioSource) was stored at 2–8 °C. Performance characteristics were as follows: detection range 31.25–2000 pg/mL, sensitivity approximately 10.4 pg/mL, intra-assay precision CV < 8%, and inter-assay precision CV < 12%.

The assay was performed according to the manufacturer’s instructions. Plates, reagents, and samples were brought to room temperature prior to analysis. Standard dilutions prepared within the assay detection range and plasma samples (100 μL) were added to the wells. After removal of the liquid, 100 μL of Detection Reagent A was added and incubated at 37 °C for 1 h. Wells were washed six times with 175 μL of wash buffer. Subsequently, 100 μL of Detection Reagent B was added and incubated at 37 °C for 30 min. Wells were washed ten times, 90 μL of substrate solution was added and incubated at 37 °C for 20 min, and the reaction was stopped with 50 μL of stop solution. Optical density was measured at 450 nm within 15 min. All samples and standards were analyzed in duplicate.
